# A non-invasive diagnostic nomogram for CHB-related early cirrhosis: a prospective study

**DOI:** 10.1038/s41598-024-66560-6

**Published:** 2024-07-03

**Authors:** Yuxia Chen, Meijuan Wei, Meng Chen, Chenyu Wu, Hongbing Ding, Xingnan Pan

**Affiliations:** Decheng Hospital of Quanzhou, Affiliated of Huaqiao University, Quanzhou, 362100 China

**Keywords:** High-frequency ultrasound, Non-invasive diagnostic, Nomogram, Chronic hepatitis B, Early liver cirrhosis, Diseases, Gastroenterology, Health care, Health occupations, Medical research, Oncology, Risk factors

## Abstract

This study aimed to construct a non-invasive diagnostic nomogram based on high-frequency ultrasound and magnetic resonance imaging results for early liver cirrhosis patients with chronic hepatitis B (CHB) which cannot be detected by conventional non-invasive examination methods but can only be diagnosed through invasive liver puncture for pathological examination. 72 patients with CHB were enrolled in this prospective study, and divided into S4 stage of liver cirrhosis and S0-S3 stage of non-liver cirrhosis according to pathological findings. Binary logistic regression analysis was performed to identify independent predictors, and a diagnostic nomogram was constructed for CHB-related early cirrhosis. It was validated and calibrated by bootstrap self-extraction. Binary logistic regression analysis showed that age (OR 1.14, 95% CI (1.04–1.27)), right hepatic vein diameter (OR 0.43, 95% CI 0.23–0.82), presence or absence of nodules (OR 31.98, 95% CI 3.84–266.08), and hepatic parenchymal echogenicity grading (OR 12.82, 95% CI 2.12–77.51) were identified as independent predictive indicators. The nomogram based on the 4 factors above showed good performance, with a sensitivity and specificity of 90.70% and 89.66%, respectively. The area under the curve (AUC) of the prediction model was 0.96, and the predictive model showed better predictive performance than APRI score (AUC 0.57), FIB-4 score (AUC 0.64), INPR score (AUC 0.63), and LSM score (AUC 0.67). The calibration curve of the prediction model fit well with the ideal curve, and the decision curve analysis showed that the net benefit of the model was significant. The nomogram in this study can detect liver cirrhosis in most CHB patients without liver biopsy, providing a direct, fast, and accurate practical diagnostic tool for clinical doctors.

## Introduction

The five-year overall survival rate for hepatic cancer is currently only 12.1%^1^. Cirrhosis was still a high-risk factor for hepatic cancer, even if active antiviral therapy was given. The diagnosis and treatment of early cirrhosis is critical for the management optimization of chronic liver disease.

In patients with chronic hepatitis B (CHB), some early cirrhosis patients cannot be detected by conventional non-invasive examinations and can only be diagnosed through invasive liver puncture for pathological examination. However, liver biopsy is an invasive procedure with operational risks. Patient compliance is often poor, and single-point sampling may lead to misdiagnosis, relying heavily on the experience of specialized pathologists. Currently, various non-invasive liver fibrosis detection technologies and scoring systems have emerged, such as transient elastography (TE), extracellular matrix components (such as hyaluronic acid, type III procollagen peptide, type IV collagen, laminin), APRI^2,3^, FIB-4^4^, INPR^5^, etc. However, due to interference factors or unsatisfactory accuracy of the results, their clinical value is limited, and they are difficult to be adopted by clinicians.

Therefore, a more efficient, non-invasive examination method is urgently needed to replace the pathological examination of liver biopsy. In this prospective study, liver biopsy pathological results were used as control to construct a non-invasive diagnostic nomogram for CHB-related early cirrhosis. Since high-frequency linear array probe (8–18 MHz) used for ultrasonography has high resolution and can clearly detect subtle changes in liver parenchyma and small intrahepatic nodules^6^, this non-invasive diagnostic nomogram based on ultrasonography using high-frequency linear array probe may improve the sensitivity and specificity for the diagnosis of CHB-related early cirrhosis.

## Patients and methods

The study protocol has been approved by the hospital ethics committee (No.: DCEC2020-158), and signed informed consent was obtained from all subjects. All methods were performed in accordance with the relevant guidelines and regulations.

### General information

**Figure 1:**
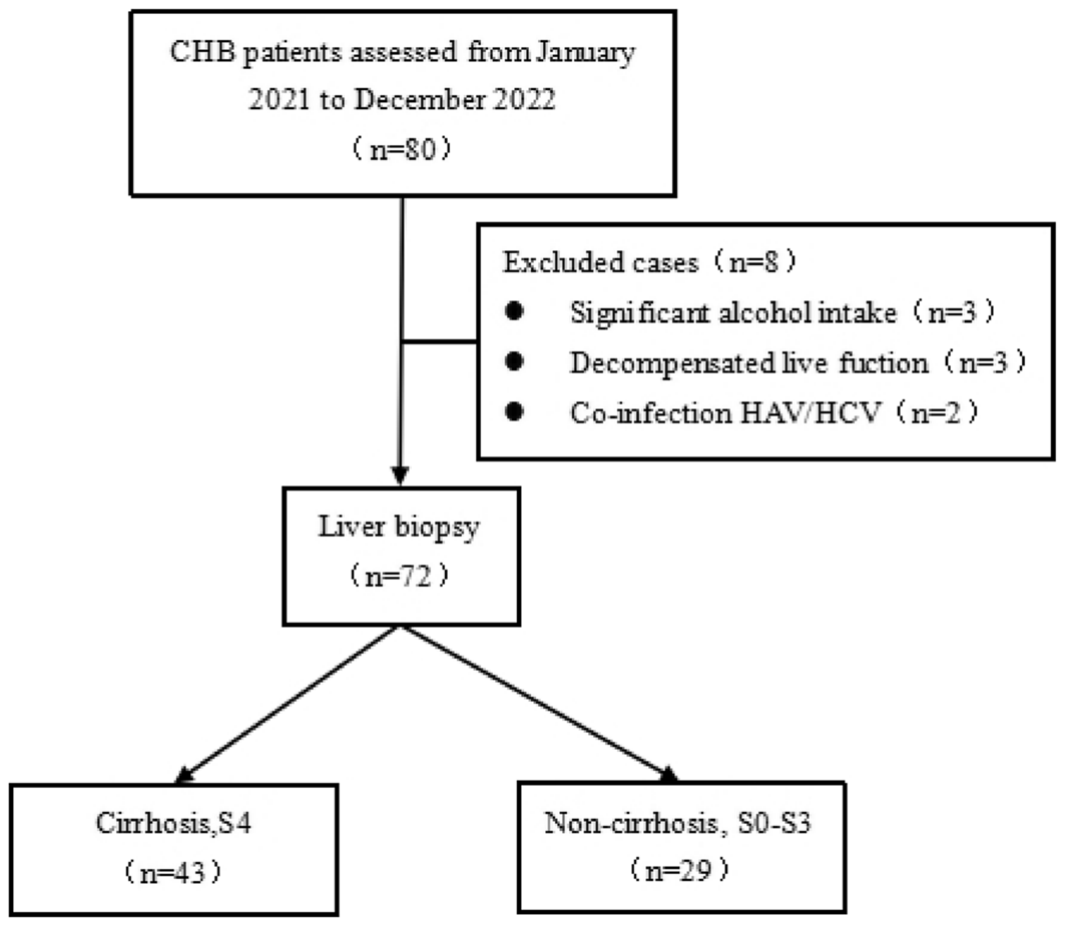
Flow chart for patient selection. CHB = chronic hepatitis B.

From July 2021 to July 2022, 80 patients with liver biopsy were prospectively recruited for CHB. Among them, 43 patients with early cirrhosis (S4) and 29 patients with non-cirrhosis (below S3) CHB met the inclusion and exclusion criteria (Fig. 1). The age of the patients ranged from 21 to 67 years, with a mean age of 40.5 years. There were 64 males and 8 females. Inclusion criteria: (1) HBsAg positive or HBV DNA positive for at least 6 months; (2) Liver function Child–Pugh A. Exclusion criteria: (1) Decompensated cirrhosis or history of any concurrent malignancy; (2) Imaging examination showed that wavy liver shape, widened hepatic fissure distance, unbalanced proportion of each lobe, and portal hypertension (such as esophageal and gastric vein varicose, azygos vein opening, etc.); (3) Pregnant or breastfeeding women; (4) Other viral hepatitis, autoimmune hepatitis, drug-induced hepatitis, alcoholic liver disease or fatty liver disease, hereditary liver disease or liver vascular disorder, and other systemic diseases. The cost of liver biopsy and other examinations in the enrolled patients was free.

### Laboratory test

Serum HBV DNA was detected by fluorescence real-time quantitative PCR (kit purchased from Guangzhou Daan Biotechnology Co., Ltd., the lower limit of detection was 20 IU/ml); HBsAg was detected by electrochemiluminescence with the Roche automatic immunoassay analyzer (kit purchased from Roche); Liver function was detected by the Mindray BS2000M automatic biochemical analyzer; the Mindray BC7300 blood cell analyzer detected platelet count, and international normalized ratio (INR) was calculated by the Mindray ExC810 automatic blood coagulation analyzer (kit provided by Mindray Company).

### LSM

According to the standard procedures, LSM test was performed using FibroTouch FT-B (Hisky Medical, China). The results were expressed using kPa, and the normal range was 1.0–80 kPa.

### Ultrasonic examination

**Figure 2:**
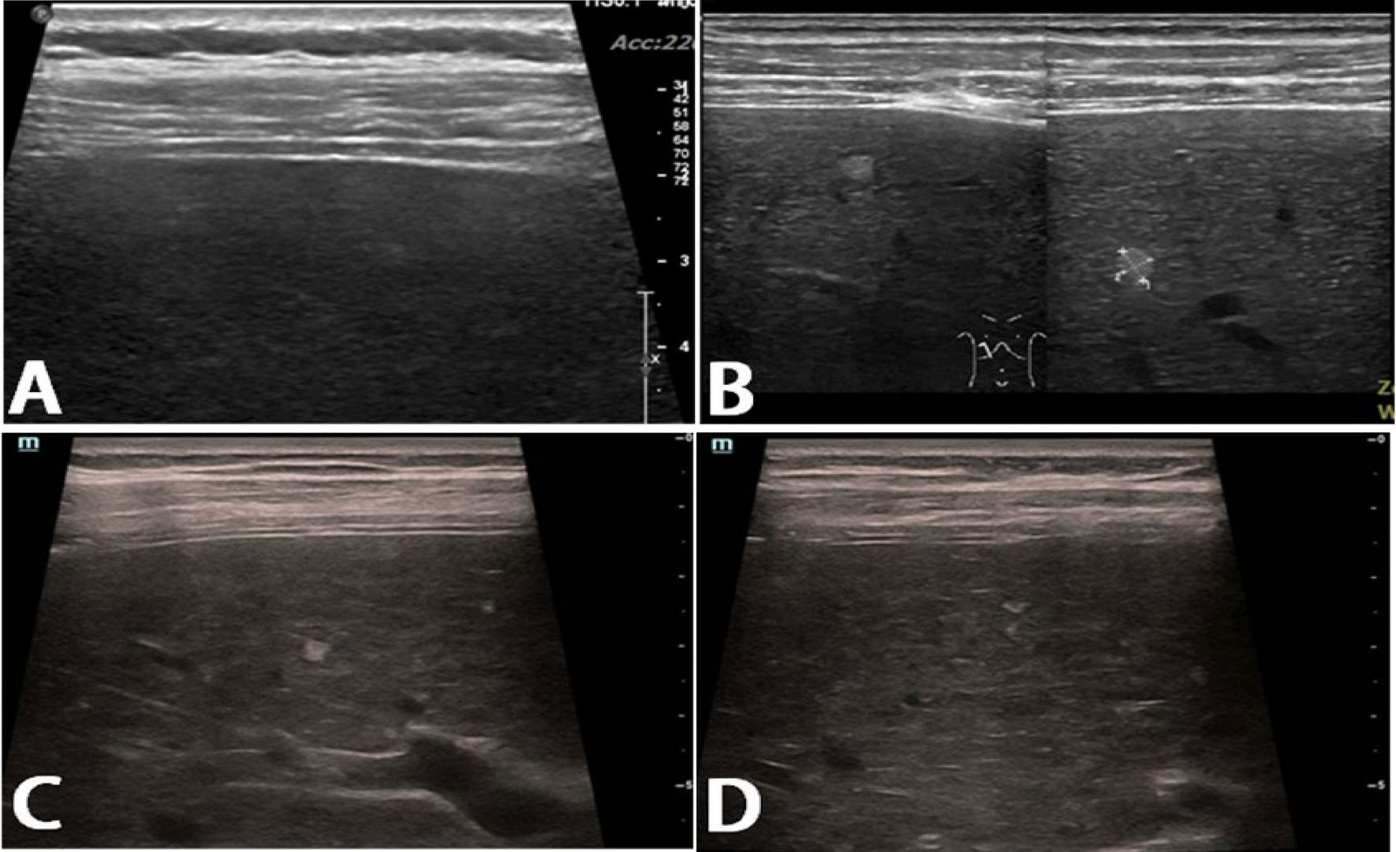
High-frequency ultrasound examination parameters and grading. (**A**) No nodule in liver (grade 0), fine granular echo in liver parenchyma (grade 0); (**B**) Scattered small hyperechoic nodules were seen in the liver, and the echo of the liver parenchyma showed coarse granules (grade 0); (**C**, **D**) The hyperechoic structure of the liver parenchyma was widened and lengthened, with an irregular shape and cord-like shape. The hypoechoic structure between the cords was irregular and widened, with strong and hypoechoic structures arranged in a staggered manner, showing a large cribriform or reticular shape. In the reticular shape, multiple hypoechoic nodules (grade 1) were scattered in the liver parenchyma.

The GE E8 intelligent ultrasonic diagnostic system was used. The Convex array probe frequency was 3.5–5.0 MHz; the linear array probe frequency was 8–12 MHz. Ultrasound examination was performed in all patients within 1 week before liver biopsy. Fasting for more than 8–12 h was required. Patients should be in a supine or left lateral decubitus position. Three relevant parameters of the liver need to be recorded: (1) Node: it can be divided into two categories according to the presence or absence of diffuse small nodules. (2) Liver parenchymal echo grading (LPEG) referred to the hyperechoic structures, which were divided into two grades, grade 0 and grade 1 (Fig. 2). (3) Portal vein flow velocity (cm/s). The procedures were performed individually by 2 sonographers, and the data were recorded.

### Magnetic resonance examination

**Figure 3:**
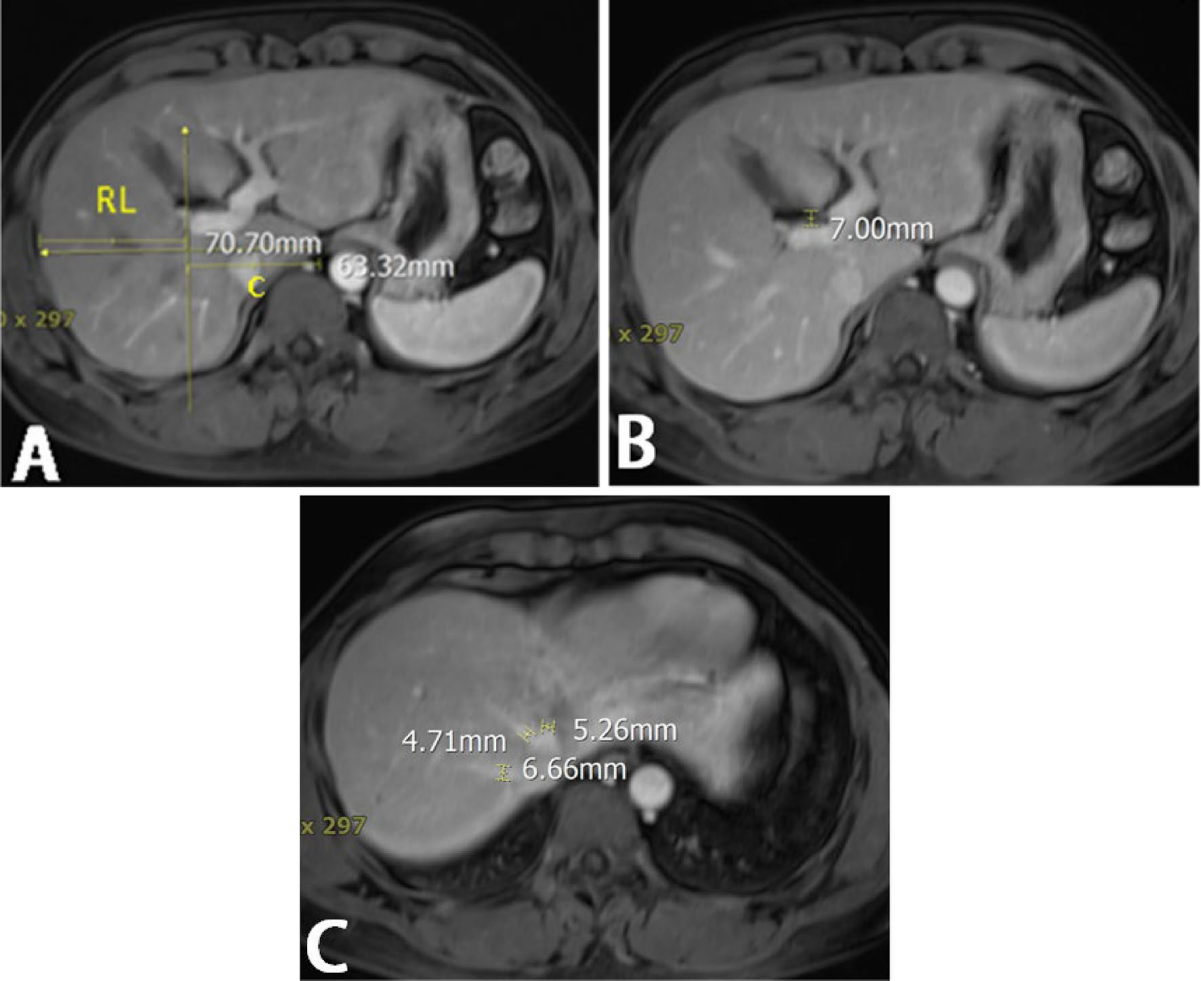
Measurement of magnetic resonance examination parameters. (**A**) hepatic fissure distance: measurement of the thickness of the fat around the hepatic hilum. In the axial view, the distance between the anterior wall of the right portal vein and the posterior margin of the medial segment of the left hepatic lobe was measured at the plane where the right portal vein appeared, with the measurement line perpendicular to the middle of the right portal venous system; (**B**) portal vein-caudate lobe distance (C), right lobe-portal distance (RL): (**C**) In the axial plane, draw a line parallel to the mid-sagittal plane through the right lateral wall of the first bifurcation of the right portal vein. Measure the distance from this drawn line to the innermost edge of the caudate lobe; RL: from the drawn line to the outer edge of the right lobe, situated between the main portal vein and the inferior vena cava; (**C**) hepatic veins on the axial image, measured at the end near the inferior vena cava.

Siemens Magneton ESSENZA 1.5 T nuclear magnetic resonance equipment was used: hepatic fissure distance, portal vein-caudate lobe distance (C), right lobe-portal distance (RL), diameter of left hepatic vein (LHV), diameter of middle hepatic vein (MHV), diameter of right hepatic venous (RHV) (Fig. 3). The images were assessed individually by two imaging physicians who were blinded to the patient information, and the data were recorded.

### Liver pathological examination

After the patient signed the informed consent, the liver puncture was performed under the guidance of ultrasound positioning, and the liver tissue was collected by percutaneous puncture with the BARD 16G biopsy needle, including at least two pieces of liver tissue which have at least 11 portal tracts, and the specimen length was about 20 mm. The liver tissue sample was stained by routine HE staining and reticular fiber staining, and liver fibrosis was scored by two pathologists according to the Guidelines for the Prevention and Treatment of Viral Hepatitis in Xi'an, 2000^7^. Liver pathological results were divided into two groups: the non-cirrhotic group (S0-S3) and the cirrhotic group (S4).

### Statistical analysis

The SPSS 26 software was used for statistical analysis, the chi-square test was used for enumeration data, and the measurement data were expressed by the median. One-way analysis of variance and independent sample t-test or a non-parametric rank sum test was used to compare the differences between the groups. The Spearman correlation coefficient was used to evaluate the correlation between grade variables. Binary Logistic regression analysis was used to screen the independent predictors of early hepatitis B cirrhosis and to establish a diagnostic model. The receiver operating characteristic (ROC) curve was drawn by the Medcalc software version 20.100, and the accuracy was calculated. The calibration curve was drawn by R language version 4.3.1. The DCA curve was drawn.

## Results

### Univariate and multivariate analysis

As shown in Fig. 1 and Table 1, 72 patients met the inclusion criteria. The results of univariate analysis showed that there were significant differences in nodule, age, LSM, CAP, HBsAg log, C, RHV, MHV, LHV, RL, LPEG, liver fissure distance, and FIB-4 between the two groups but not in gender, PLT, RL, AST, ALT, HBV DNA, APRI, INPR and portal vein flow velocity (Table 1). However, only age, RHV, nodule, and LPEG were identified as independent factors associated with the CHB-related early cirrhosis (Table 2) by multivariable analysis.

**Table 1:** Univariate analysis of the early hepatitis B cirrhosis-related clinical indicators.

Clinical indicators	Classification	Non-cirrhotic group (n = 29)	Cirrhosis group (n = 43)	χ^2^/*t*/*z* value	*p*-value
Gender [n (%)]	Male	27 (93.10)	37 (86.05)	0.87	0.35
	Female	2 (6.90)	6 (13.95)		
Nodule [n (%)]	Without nodules	26 (89.66)	14 (32.56)	22.87	0.00**
	With nodules	3 (10.34)	29 (67.44)		
Liver parenchymal echo grading	Grade 0	22 (75.86)	10 (23.26)	19.41	0.00**
	Grade 1	7 (24.14)	33 (76.74)		
Age (year)		35.79 ± 7.48	43.67 ± 9.59	− 3.73	0.00**
PLT (× 10^9^/L)		204.90 ± 51.82	187.70 ± 54.24	1.34	0.18
INR		0.95 ± 0.08	1.01 ± 0.11	− 2.43	0.02*
Controlled attenuation parameter (CAP, dB/m)		229.24 ± 20.24	243.26 ± 29.46	− 2.39	0.02*
HBsAglog		3.00 (2.00, 4.00)	3.00 (2.00, 3.00)	− 2.39	0.02*
Diameter of right hepatic vein (RHV, mm)		7.54 ± 1.63	5.40 ± 1.52	5.68	0.00**
Diameter of middle hepatic vein (MHV, mm)		6.53 ± 1.27	5.50 ± 1.37	3.22	0.00**
Diameter of Left hepatic vein (LHV, mm)		6.61 ± 2.17	5.20 ± 1.17	3.22	0.00**
Portal vein-caudate lobe distance (C, mm)		62.56 ± 12.50	69.48 ± 9.95	− 2.61	0.01*
Right lobe-portal distance (RL, mm)		66.89 ± 7.92	63.99 ± 7.77	1.54	0.13
AST (U/L)		28.20 (22.00, 52.20)	39.30 (25.90, 71.90)	− 0.53	0.60
ALT (U/L)		41.100(27.60, 79.60)	38.65 (25.90, 73.30)	− 0.42	0.68
HBV DNA (IU/ml)		10,200.00 (500.00, 67,300,000.00)	2437.00 (500.00, 2,850,000.00)	− 0.89	0.37
Liver stiffness measurement (LSM, kPa)		8.93 ± 3.09	11.79 ± 5.13	− 2.679	0.009**
Portal vein flow velocity (cm/s)		20.50 (18.00, 25.00)	20.00 (18.00, 23.40)	− 0.301	0.764
Hepatic fissure distance (mm)		8.38 ± 4.22	10.62 ± 4.48	− 2.134	0.036*
APRI		0.38 (0.30, 0.60)	0.45 (0.30, 0.90)	− 0.96	0.34
FIB-4		0.82 (0.60, 1.00)	1.13 (0.80, 2.10)	− 2.08	0.04*
INPR		0.49 (0.40, 0.60)	0.52 (0.40, 0.70)	− 1.87	0.06

**p < 0.001 **p < 0.005 * *p* < 0.05 * *p* < 0.01.

**Table 2:** Multivariate analysis of clinical indicators of CHB-related early cirrhosis.

Variables	Beta	S.E	Z	OR (95%CI)	*p*-value
Age (years)	0.13	0.57	5.12	1.14(1.02–1.27)	0.02*
Diameter of right hepatic vein (RHV, mm)	− 0.84	0.33	6.58	0.431(0.23–0.82)	0.01*
Nodules					
With nodules	3.47	1.08	10.28	31.98 (3.84–266.08)	0.00**
Without nodules					
Liver parenchymal echo grading					
Grade 0					
Grade 1	2.55	0.92	7.72	12.82 (2.12–77.51)	0.01**

**p < 0.001 **p < 0.005 * *p* < 0.05* *p* < 0.01.

### Establishment of a prediction model for early cirrhosis in CHB:

**Figure 4:**
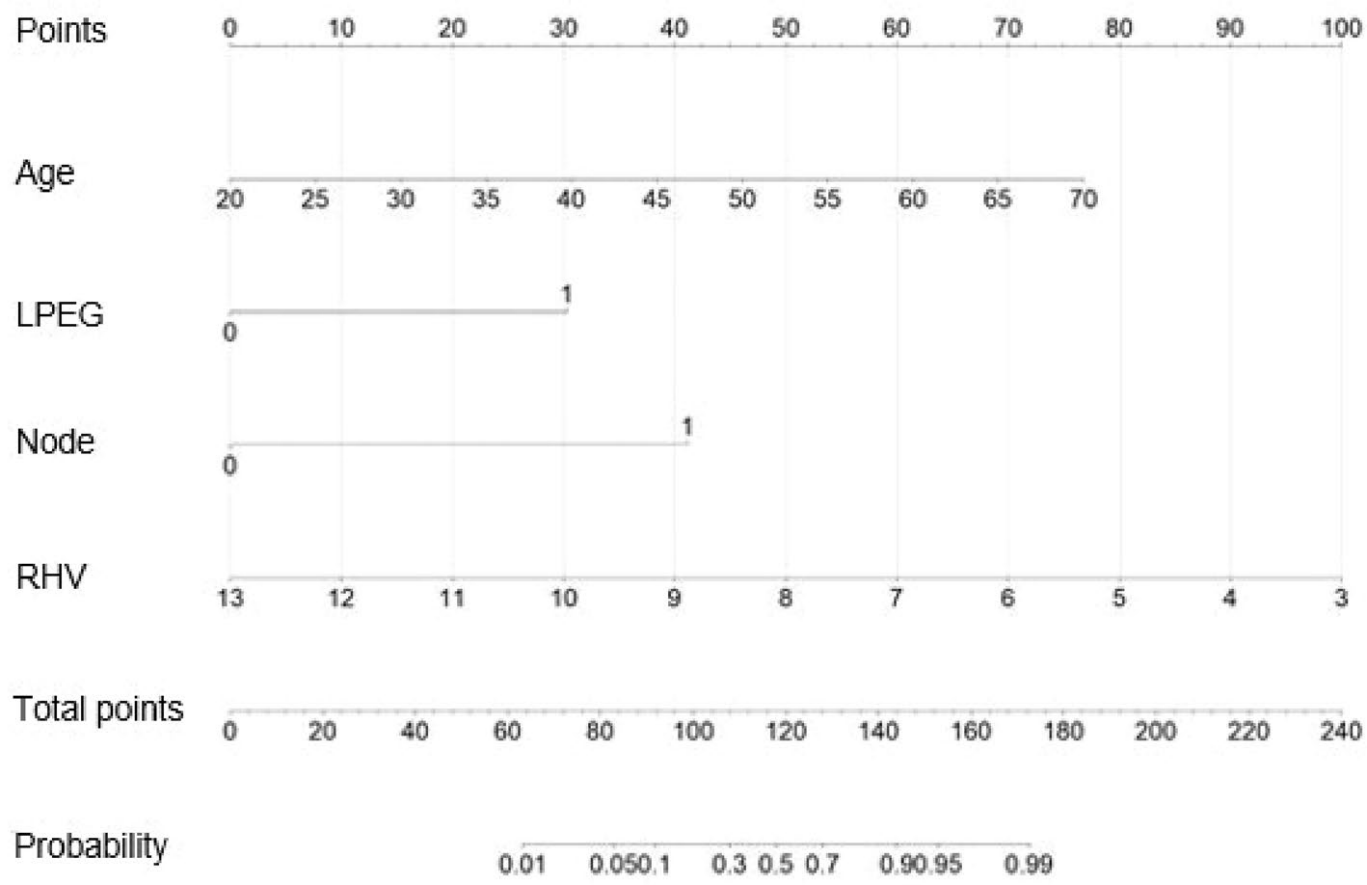
Diagnostic nomogram for CHB-related early cirrhosis.

Based on the RMS software package of the R software, the independent predictors screened by the above multivariate analysis were included to construct the diagnostic nomogram for CHB-related early cirrhosis. As shown in Fig. 4, the four predictors on the left are Age, RLV, Node and LPEG from top to bottom, and according to the scores of different factors corresponding to the actual clinical data of the patients, the scores of the 4 predictors were added.

### Validation of the prediction model

**Figure 5:**
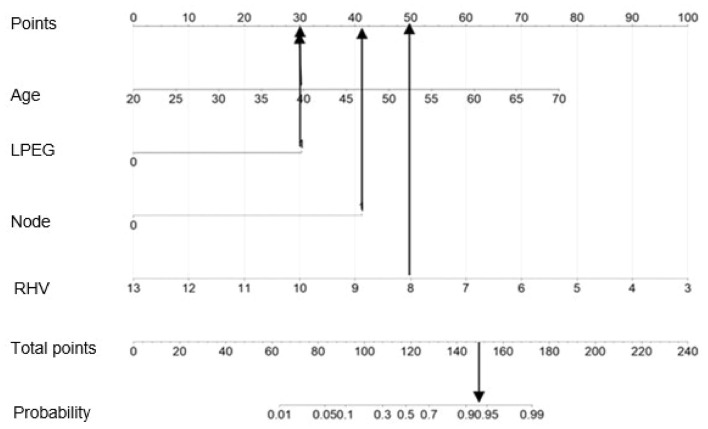
Application of the diagnostic nomogram for CHB-related early cirrhosis.

For example, a patient receives serum chemistry tests, age 40 years old, liver parenchyma echo grade 1 Doppler ultrasound showed multiple nodules in the liver, diameter of right hepatic vein8mm, as shown in Fig. 5. Based on the prediction model, the patient would score 30 points for age, 30 points for parenchymal echo grade, 41 points for nodules, and 50 points for right hepatic vein diameter. The total score would be 151, which, when plotted vertically downward, added up to about 93%, indicating that the predictive probability of the patient being diagnosed with early cirrhosis would be 93%.

### ROC Curve of nomogram prediction model

**Figure 6:**
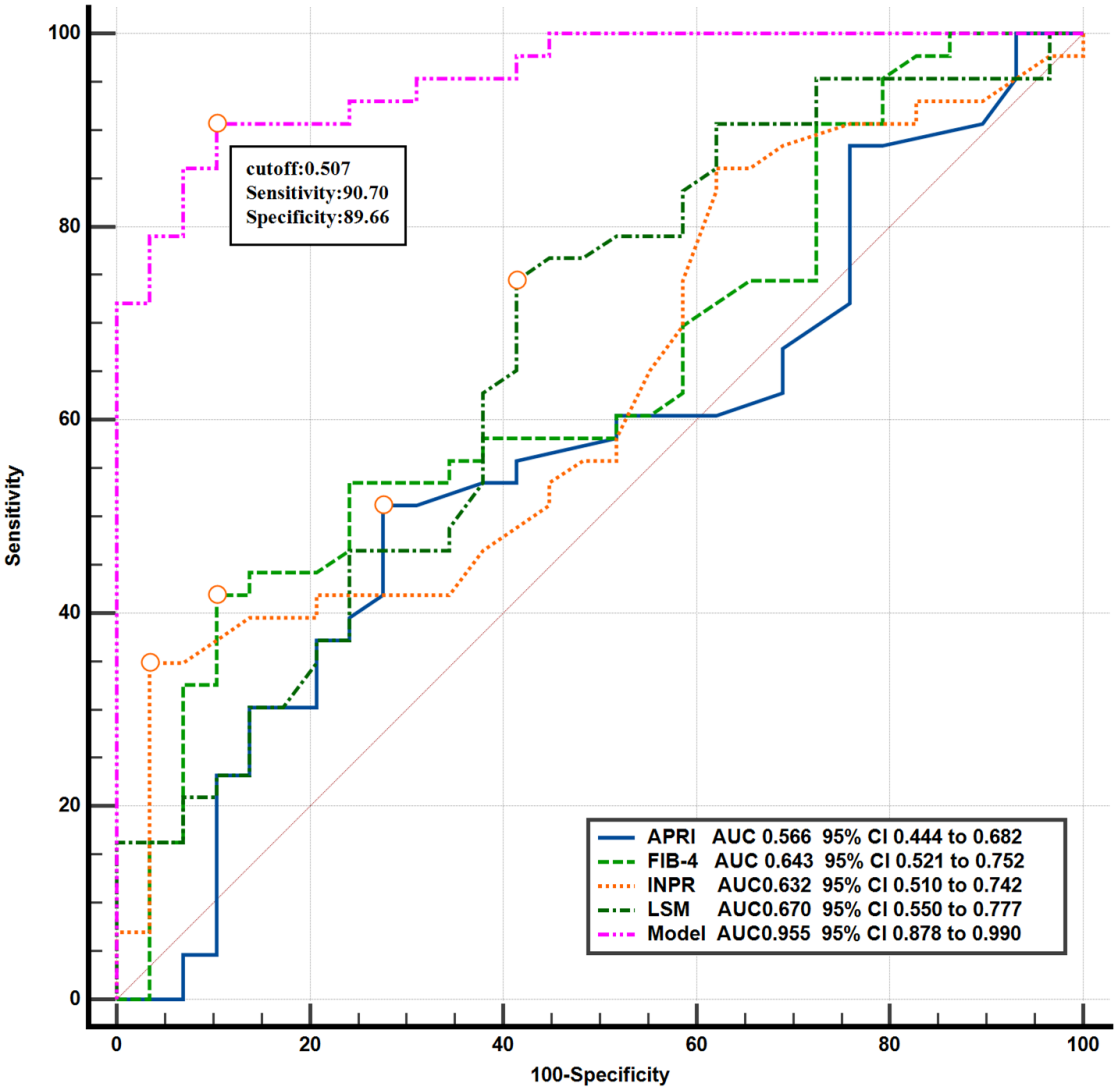
Comparison of the ROC curve between the prediction model and the APRI, FIB-4, INPR scores, and LSM.

The ROC curve of the diagnostic nomogram is shown in Fig. 6. The AUC was 0.96, the optimal threshold was 0.51, the sensitivity was 0.91, and the specificity was 0.90. The accuracy of the diagnostic model was 0.88. The AUC value of the diagnostic nomogram is 0.96 > 0.70, which indicates that the prediction accuracy is good. The score over 0.51 predicted early cirrhosis. The corresponding sensitivity, specificity, and 95% CI were 90.70%, 89.66%, and 0.89–0.990, respectively. The Youden index J was 0.80. The nomogram showed higher AUC values than the APRI, FIB-4, INPR scores, and LSM, indicating that it outperformed these scoring systems in terms of predictive capability.

### C-index and calibration curves were used to validate the prediction model

**Figure 7:**
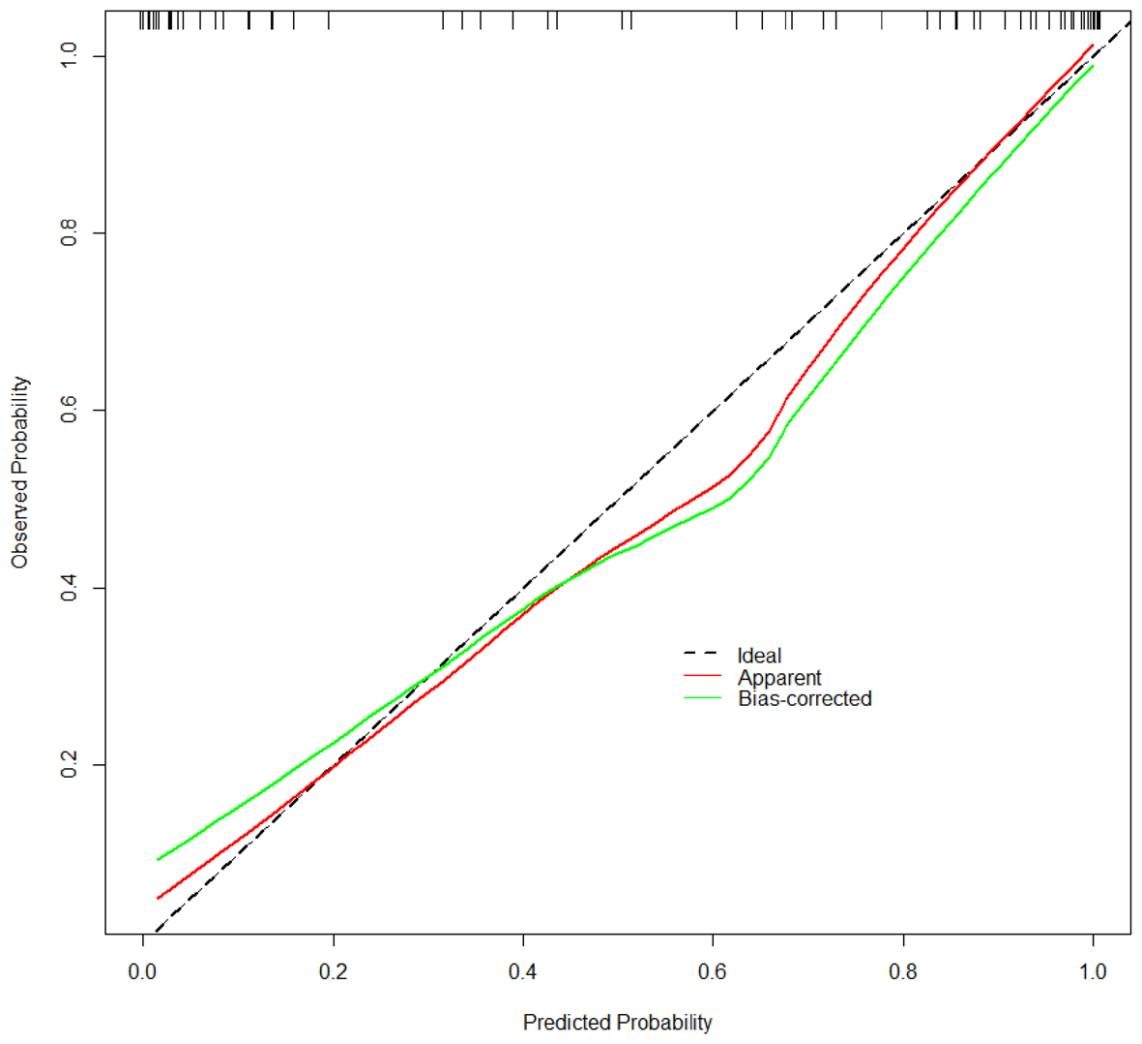
Calibration curve of prediction model.

The C-index of the prediction model was 0.96, and the adjusted C-index calculated by the Bootstrap self-extraction method was 0.94, which indicated that the prediction model had good accuracy. At the same time, a calibration curve was drawn, as shown in Fig. 7, and the dotted line represents the ideal curve corresponding to the predicted result and the actual result. The calibration curve fitted well with the ideal curve, indicating that the diagnostic nomogram had a good predictive capability for the diagnosis of early liver cirrhosis.

### DCA curve of the diagnostic nomogram for CHB-related early cirrhosis

**Figure 8:**
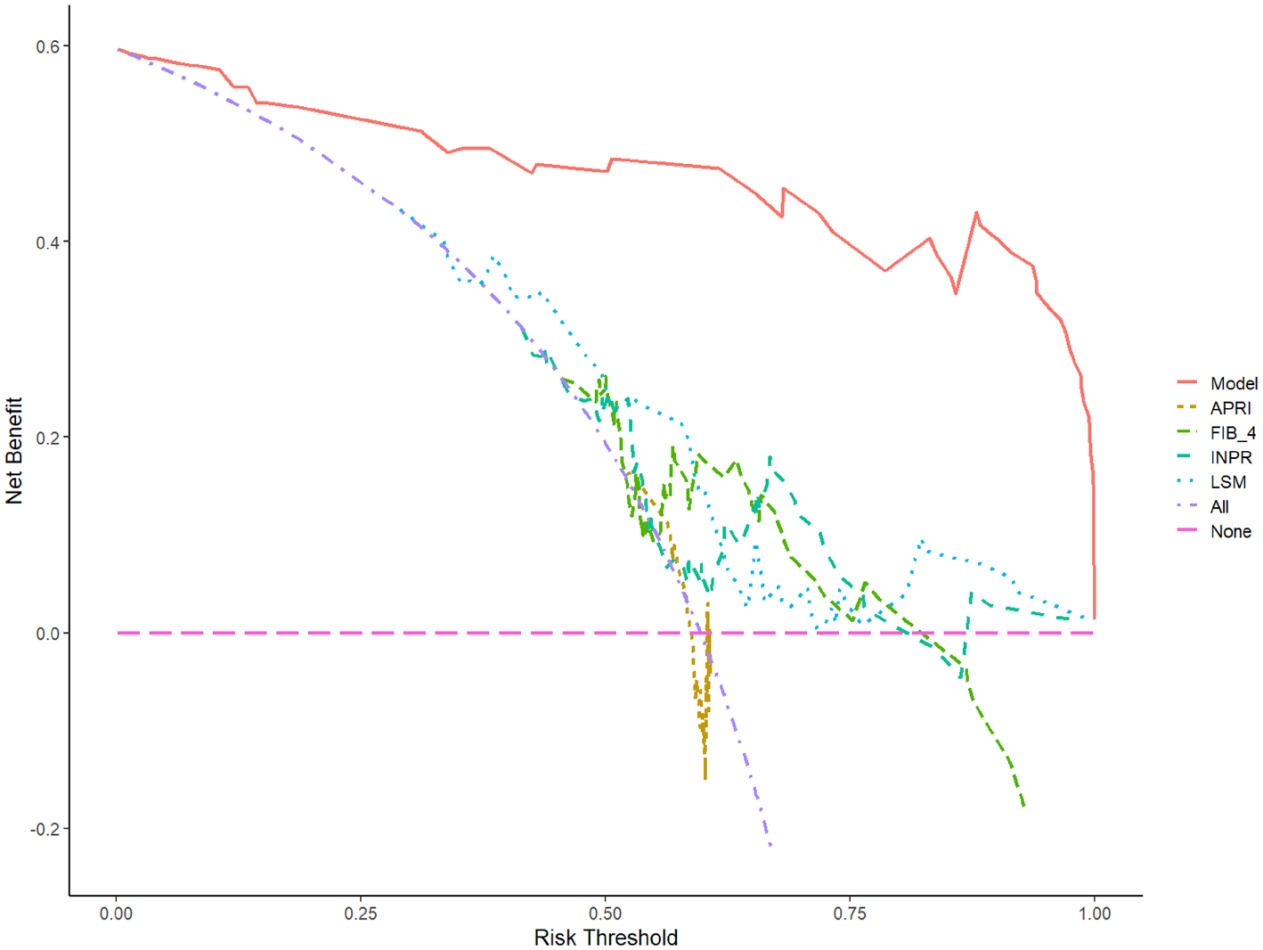
Comparison diagram of the DCA curves of our prediction model and APRI, FIB-4, INPR, LSM.

The purple Oblique dashed line and the pink Horizontal line represent two extreme cases. The pink Horizontal dashed line indicates that none of the patients had cirrhosis and were not diagnosed as cirrhosis. In this case, there was no diagnostic benefit. The purple oblique dashed line indicates that all patients were diagnosed as cirrhosis and, therefore, all accepted the diagnosis. Red represents the model. Generally speaking, the farther the curve is from the two extreme cases, the better. As shown in Fig. 8, the net benefit of this model was more obvious than others in the threshold range of 0–99%. The diagnostic nomogram of this study is clinically valuable.

## Discussion

Early cirrhosis is not easy to be detected directly by the existing medical instruments. Liver pathological examination is the "gold standard" for the diagnosis of early cirrhosis. While liver biopsy is an invasive examination, it is rejected by most patients. Transient elastography (TF) war used to determine the status of liver fibrosis by detecting the liver stiffness measurement (LSM), which can be influenced by factors such as the patient's BMI, age, diabetes, alcohol consumption, diet, ALT, TBIL, leading to potential errors. According to the updated Expert Consensus on the Diagnosis of Liver Fibrosis by Transient Elastography in 2018, cirrhosis was considered for CHB patients with normal bilirubin and ALT < 5 × ULN at LSM 17.00 kPa, and cirrhosis was considered for CHB patients with normal bilirubin and ALT at LSM 12.00 kPa^8^. Clinically, some patients still had missed diagnosis of cirrhosis. At present, APRI, FIB-4, and INPR are relatively simple and valuable in clinical application, but their clinical utility rate is low. The main objective of this study was to identify early cirrhosis. In our research, it was revealed that age, diameter of right hepatic vein (RHV), presence or absence of nodule (Node), and liver parenchymal echo grading (LPEG) were independent risk factors of CHB-related early cirrhosis. These four independent risk factors were used to construct a diagnostic nomogram. After verification, it was found that the diagnostic nomogram has a better prediction effect and that its prediction capability is more accurate than the APRI, FIB-4, INPR scores, and LSM.

### Predictive value of each index for CHB-related early cirrhosis

#### Predictive value of age in early liver cirrhosis

The annual incidence of cirrhosis in patients with CHB without antiviral therapy is 2% to 10%. The risk factors of the host include older age, male sex, > 40 years of age at the time of HBeAg seroconversion, and abnormal ALT^9^. The immune response caused by HBV leads to liver cell injury, inflammation, and necrosis, and the persistence or recurrence of inflammation and necrosis is an important factor for chronic HBV infection to progress to cirrhosis or even HCC. These lesions take a long time to develop, and the risk of cirrhosis increases significantly with age. Many recent studies and published foreign guidelines have pointed out that age > 30 is an independent risk factor for disease progression^10,17–25^.

#### Predictive value of right hepatic vein diameter (RHV) in early liver cirrhosis

From a pathological perspective, the whole-liver diffuse pseudo-lobular structure in cirrhosis alters the liver's microcirculatory system and subsequently alters hemodynamics. During this process, changes in the hepatic venous system are particularly significant. According to Wanless, in the cirrhotic or necrotic liver, proliferating fibroblasts produce a large amount of extracellular matrix, hepatic veins are compressed, and the lobular structure is severely disordered, which also prevents new hepatocytes from filling the collapsed lobules^11^. Therefore, without a proper structural framework (basement membrane scaffold), hepatocyte regeneration is blocked, and normal liver structure cannot be produced. The expanding regenerative nodule compresses the hepatic vein, resulting in congestion of the hepatic sinusoid, necrosis of the hepatocytes, and a new regenerative cycle. In a study of the correlation between cirrhosis and vessel size by Zhang et al., it was found that a RHV diameter less than 7 mm was probable cirrhosis (sensitivity 88%, specificity 85%), and that less than 5 mm was highly suspicious cirrhosis (specificity 99%)^12^. The subjects in the above literature were patients with advanced cirrhosis, while the subjects in this present study were patients with early cirrhosis. There were significant differences in the three independent variables of left, middle, and right hepatic vein diameters (LHV, MHV, RHV) through univariate screening (*p* = 0.00, 0.00, 0.00, respectively). Multivariate regression analysis showed that only RHV was significantly associated with early cirrhosis (*p* = 0.01). In addition, the RHV in this study was measured by magnetic resonance plain scan, not by ultrasound imaging, because the value measured by the latter is easily affected by fatty liver and intestinal gas interference. In contrast, magnetic resonance has the characteristics of fat suppression and no intestinal gas interference, so it can more accurately measure RHV.

#### Predictive value of node in early cirrhosis

Liver cirrhosis is characterized by pathological changes of hepatocyte necrosis, connective tissue hyperplasia, and hepatocyte regeneration. Fibrosis in liver diseases begins in the portal area. It gradually expands to the periphery until the formation of fibrous septa, and finally accompanied by the formation of lobular structure disorder, pseudo lobules, and liver regeneration nodules^13^.

These structural and pathological changes provide a theoretical basis for the exploration of high-frequency ultrasound in the diagnosis of liver fibrosis and the discovery of small nodules. Wanless proposed the concept of "liver repair complex (HRC)" in liver cirrhosis-related literature, in which "small regenerative nodules" are directly manifested as the aggregation of new hepatocytes in histopathology, which is called "budding". In liver diseases, there are no mature hepatocytes that can regenerate in the necrotic collapsed areas, and hepatocyte "buds" derived from precursor cells form and mature to fill the collapsed parenchyma area. All patients with cirrhosis^14^ had regenerative nodules (RN) throughout the liver, and the MR display rate was 50%. Common diffuse liver diseases that need to be distinguished from RN, such as fatty nodules, are mainly caused by T1 hyperintensity in fat. The best way to display this is with both in-phase and out-of-phase imaging, which shows increased signal intensity in the in-phase and decreased signal intensity in the out-of-phase, with clear subtraction. Decreased tissue perfusion is also a possible cause of fatty nodules. Ultrasound imaging can help to detect small intrahepatic nodules more sensitively through high-frequency probes, and smaller nodules that cannot be displayed by MR may be easily identified by ultrasound. At present, a large number of literatures also support that the application of high-frequency probes to detect liver micro-nodules can further improve the diagnostic accuracy of cirrhosis. However, the reasons for the inconsistency between ultrasound and pathology include: (1) If the regenerative nodule is large, the sample is just located in the nodule, or the complete fibrous cord is not taken, then the ultrasound results will be more severe than the pathological results, resulting in a case of cirrhosis diagnosed by ultrasound, but not supported by the pathological results. (2) Diffuse hyperechoic nodules in the liver parenchyma, common in early cirrhosis nodules, can also be seen in fat nodules. Therefore, it needs to be further combined with other indicators to be judged. In this study, the presence or absence of nodules was used as a dichotomous independent variable. After multivariate regression analysis, it was significantly associated with early cirrhosis (p-value 0.001), which had a high predictive value.

#### Predictive value of liver parenchymal echo grading (LPEG) in early cirrhosis

The echo shape of liver parenchyma is a commonly used parameter in ultrasound imaging, which can improve the accuracy of diagnosis of early cirrhosis. Some studies use the high-resolution characteristics of high-frequency ultrasound to grade the echo of liver parenchyma. The grades were established according to the morphological changes based on combining ultrasound imaging and histopathology^15^, which overcomes the artificial factors and the issue of vague description in the previous understanding of liver parenchyma echo by low-frequency ultrasound. In this study, high-frequency ultrasound was used to observe the hyperechoic morphological changes of liver parenchyma and to record the morphological changes of liver parenchyma in different stages of liver fibrosis. There are two main reasons for the inconsistency between the results and the pathology: (1) Affected by the fatty degeneration of hepatocytes, the liver echo is enhanced, which covers up the fibrosis of the liver tissue itself, and at the same time, it also affects the acoustic impedance difference between the portal area and the hepatic lobule, thus affecting the judgment of the structure of the portal area^16^. (2) The location of high-frequency ultrasound observation is not necessarily the location of liver tissue sampling. In this study, early cirrhosis was taken as the research object, and it was simpler and more convenient to divide it into two grades; the results were easy to obtain, and the clinical practicability was higher. Binary logistic regression combined with other independent variables could reduce the error caused by the above factors and improve the prediction sensitivity. As can be seen from the results, the p-value of liver parenchymal echo grading was 0.005, which was significantly different and showed a significant correlation with CHB-related early cirrhosis in multivariate regression analysis.

### Diagnostic value of a nomogram for CHB-related early cirrhosis

In this study, univariate analysis and multivariate binary logistic regression analysis revealed that age, right hepatic vein diameter (RHV), presence or absence of nodule (Node) and liver parenchymal echo grading (LPEG) were independent risk factors in CHB-related early cirrhosis. Using the above indicators to construct a nomogram, we can guide clinicians to judge CHB-related early cirrhosis more quickly, accurately, and non-invasively and strive for early and reasonable clinical intervention.

The ROC curve showed that the AUC value was 0.96, and the optimal cut-off value was 0.51. The results above the optimal cut-off point of 0.51 could predict early cirrhosis. The corresponding sensitivity, specificity, and 95% CI were 90.70%, 89.66%, and 0.88–0.99, respectively. At the same time, the ROC curves of the APRI, FIB-4, INPR scores and LSM were compared, and the AUC values were calculated (0.57, 0.64, 0.63, and 0.67 respectively), which confirmed that the model in this study had better predictive capability. In terms of validation, the consistency index calculated by the Bootstrap self-extraction method was 0.94, which indicates that the prediction model has good accuracy. The calibration curve fitted well with the ideal curve, indicating that the diagnostic nomogram had a good predictive effect in the diagnosis of early cirrhosis. The sensitivity and specificity of the prediction model were high, which confirmed that the prediction model had a high clinical application value. Compared with the DCA curves of APRI, FIB-4, INPR, and LSM, the net benefit rate of our model was significantly better than that of the latter three, which confirms that the prediction model of this study has practical value in clinical practice. In the process of clinical diagnosis, early cirrhosis should be predicted according to specific clinical data and actual situation, and accurate judgment should be made by using different scoring criteria and nomograms.

The ROC curve showed that the AUC value was 0.955, and the accuracy was 0.875. The diagnostic efficacy of this model was much higher compared to other non-invasive in vitro diagnostic models reported for the patients.

### Limitations and deficiencies

Although the prediction model constructed in this study has been proven to be clinically useful, it still has shortcomings and limitations. Firstly, this study was validated by the Bootstrap self-extraction method, and further external validation and analysis are needed for higher accuracy and completeness. Secondly, the number of cases in this study was small, and the results need to be confirmed by studies with a larger sample size. Thirdly, at present, other chronic liver diseases, such as HCV, autoimmune hepatitis, drug-induced hepatitis, alcoholic liver disease or fatty liver disease were not investigated in this study, and our findings remain to be verified in other chronic liver diseases (Supplementary Information file 1).

## Conclusion

Age, RHV, Node, and LPEG are independent risk factors for the diagnosis of CHB-related early cirrhosis. The non-invasive nomogram based on high-frequency ultrasonography can achieve accurate diagnosis of CHB-related early cirrhosis. In this prospective study, we constructed a nomogram that showed a better diagnostic accuracy in predicting histological cirrhosis. Based on our results, it was found that biopsy can be avoided in low- and high-risk groups. We hope that other researchers may assess the reproducibility of nomograms for noninvasive diagnosis of cirrhosis in independent populations with different clinical backgrounds.

### Supplementary Information

Supplementary Information.

## Data Availability

The datasets used and/or analysed during the current study available from the corresponding author on reasonable request.
